# Nucleoside 5′-Phosphoramidates Control the Phenylpropanoid Pathway in *Vitis vinifera* Suspension-Cultured Cells

**DOI:** 10.3390/ijms222413567

**Published:** 2021-12-17

**Authors:** Małgorzata Pietrowska-Borek, Jędrzej Dobrogojski, Anna Maria Wojdyła-Mamoń, Joanna Romanowska, Justyna Gołębiewska, Sławomir Borek, Koichi Murata, Atsushi Ishihara, Maria Ángeles Pedreño, Andrzej Guranowski

**Affiliations:** 1 Department of Biochemistry and Biotechnology, Poznań University of Life Sciences, 60-632 Poznań, Poland; jedrzej.dobrogojski@up.poznan.pl (J.D.); anna.wojdyla@up.poznan.pl (A.M.W.-M.); guranow@up.poznan.pl (A.G.); 2 Department of Nucleoside and Nucleotide Chemistry, Institute of Bioorganic Chemistry, Polish Academy of Sciences, 61-704 Poznań, Poland; joarom@ibch.poznan.pl (J.R.); jgolebiewska@ibch.poznan.pl (J.G.); 3 Department of Plant Physiology, Adam Mickiewicz University Poznań, 61-614 Poznań, Poland; borek@amu.edu.pl; 4 Graduate School of Agriculture, Tottori University, Tottori 680-8553, Japan; voba.scan24@gmail.com; 5 Department of Life and Environmental Agricultural Sciences, Tottori University, Tottori 680-8553, Japan; aishihara@tottori-u.ac.jp; 6 Department of Plant Biology, Faculty of Biology, University of Murcia, 30100 Murcia, Spain; mpedreno@um.es

**Keywords:** grape, uncommon nucleotides, signaling molecules, stilbenes, lignin

## Abstract

It is known that cells contain various uncommon nucleotides such as dinucleoside polyphosphates (Np_n_N’s) and adenosine 5′-phosphoramidate (NH_2_-pA) belonging to nucleoside 5′-phosphoramidates (NH_2_-pNs). Their cellular levels are enzymatically controlled. Some of them are accumulated in cells under stress, and therefore, they could act as signal molecules. Our previous research carried out in *Arabidopsis thaliana* and grape (*Vitis vinifera*) showed that Np_n_N’s induced the expression of genes in the phenylpropanoid pathway and favored the accumulation of their products, which protect plants against stress. Moreover, we found that NH_2_-pA could play a signaling role in *Arabidopsis* seedlings. Data presented in this paper show that exogenously applied purine (NH_2_-pA, NH_2_-pG) and pyrimidine (NH_2_-pU, NH_2_-pC) nucleoside 5′-phosphoramidates can modify the expression of genes that control the biosynthesis of both stilbenes and lignin in *Vitis vinifera* cv. Monastrell suspension-cultured cells. We investigated the expression of genes encoding for phenylalanine ammonia-lyase (*PAL1*), cinnamate-4-hydroxylase (*C4H1*), 4-coumarate:coenzyme A ligase (*4CL1*), chalcone synthase (*CHS1*), stilbene synthase (*STS1*), cinnamoyl-coenzyme A:NADP oxidoreductase (*CCR2*), and cinnamyl alcohol dehydrogenase (*CAD1*). Each of the tested NH_2_-pNs also induced the expression of the *trans*-resveratrol cell membrane transporter *VvABCG44* gene and caused the accumulation of *trans*-resveratrol and *trans*-piceid in grape cells as well as in the culture medium. NH_2_-pC, however, evoked the most effective induction of phenylpropanoid pathway genes such as *PAL1*, *C4H1*, *4CL1*, and *STS1*. Moreover, this nucleotide also induced at short times the accumulation of *N*-benzoylputrescine (BenPut), one of the phenylamides that are derivatives of phenylpropanoid and polyamines. The investigated nucleotides did not change either the lignin content or the cell dry weight, nor did they affect the cell viability throughout the experiment. The results suggest that nucleoside 5′-phosphoramidates could be considered as new signaling molecules.

## 1. Introduction

**Figure 1:**
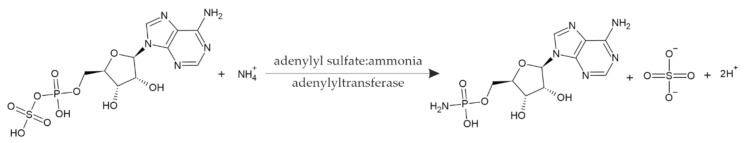
Scheme of the reaction catalyzed by adenylyl sulfate:ammonia adenylyltransferase (EC 2.7.7.51).

Continuing our long-lasting studies on uncommon nucleotides, over a decade ago [1,2,3,4,5,6], we began to investigate the biochemistry of a rather neglected nucleotide, adenosine 5′-phosphoramidate (NH_2_-pA), since it appeared to be a very good substrate of Fhit (for fragile histidine triad) proteins [7]. Much earlier, this compound was detected among cellular nucleotides purified from the alga *Chlorella pyrenoidosa* [8]. It is known that in various organisms including plants, NH_2_-pA can be synthesized [5,9] and degraded [7,10,11] by various enzymes, and it is considered as an enzymatic mechanism controlling the concentration of this nucleotide in cells. Its synthesis proceeds according to the reaction SO_4_-pA + NH_4_^+^ → NH_2_-pA + SO_4_ ^2^^−^ + 2H^+^ catalyzed by adenylyl sulfate:ammonia adenylyltransferase (EC 2.7.7.51) (Figure 1). This activity was found in the algae *Chlorella pyrenoidosa* and *Euglena gracilis*, the amoeba *Dictyostelium discoideum*, the bacterium *Escherichia coli*, and the higher plants *Hordeum vulgare*, *Spinacia oleracea* [8], and *Lupinus luteus* [5]. In the latter organism, this transferase activity proved to be an inherent property of dinucleoside triphosphatase, the Fhit protein [5]. So far, various enzymes have been shown to catalyze the degradation of NH_2_-pA, in most cases by hydrolysis to ammonia and AMP [10,11,12,13,14,15], and in a few cases to ammonia and ADP by phosphorolysis [14]. Interestingly, Fhits, regardless of their origin, are able to catalyze both the synthesis and cleavage of NH_2_-pA [5].

**Figure 2:**
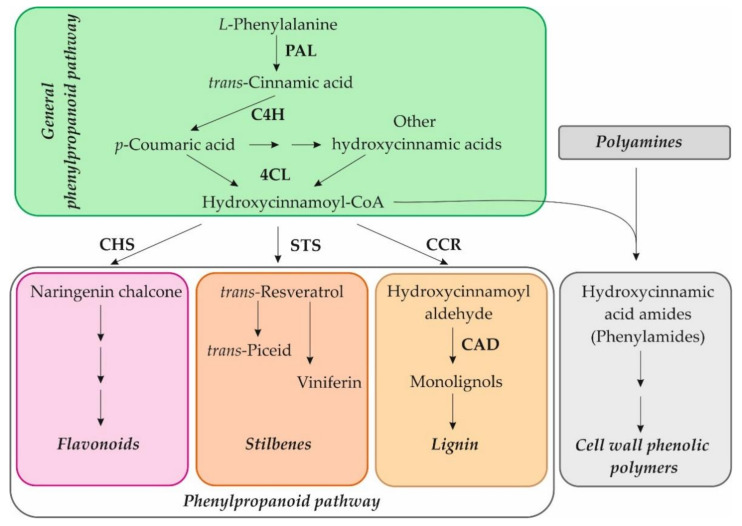
Scheme of the phenylpropanoid pathway and connection to the phenylamide metabolism. PAL, phenylalanine ammonia-lyase; C4H, cinnamate-4-hydroxylase; 4CL, 4-coumarate:CoA ligase; CHS, chalcone synthase; STS, stilbene synthase; CCR, cinnamoyl-CoA reductase; CAD, cinnamyl alcohol dehydrogenase.

Our recent studies on in vitro cultivated *Arabidopsis* seedlings showed that exogenous NH_2_-pA induced the expression of genes of the general phenylpropanoid pathway such as *PAL1, PAL2*, *PAL3*, *PAL4*, *C4H, 4CL1*, *4CL2,* and *4CL3*. Moreover, it was also observed that induction of *CCR2, CHS,* and *ICS2* expression caused the accumulation of lignins, anthocyanins, and salicylic acid, respectively [4], which protect cells against various types of stresses. Other compounds that are involved in plant defenses against abiotic and biotic stresses are phenylamides, also termed as phenolamides or hydroxycinnamic acid amides [16]. The phenylamides arise from phenolic moieties, hydrocinnamic and benzoic acids, covalently linked through amide bonds to an aromatic monoamine or an aliphatic polyamine. Their synthesis is positioned at the crossroads of the phenylpropanoid pathway and the metabolism of amines [16] and can be used in the cross-linking of cell wall components in plants (Figure 2) [17,18]. An elevated concentration of phenylamides has been reported in a wide range of plant species, and it can play a protective role against biotic stresses [19,20,21]. This is why we decided to check whether the NH_2_-pNs also affect the metabolism of those compounds.

Although it is not known whether any of the NH_2_-pNs accumulates in response to environmental stresses, according to our earlier observation of the induction of the phenylpropanoid pathway in *Arabidopsis thaliana* seedlings by NH_2_-pA [4], it seems plausible that biotic and abiotic environmental factors do affect the accumulation of this nucleotide, the putative regulatory molecule. The signaling transduction pathways underlying both abiotic and biotic stresses mediating the regulation of cellular responses are still intensively studied by many researchers. One of the defense strategies in higher plants under abiotic and biotic stresses is an activation of the phenylpropanoid pathway [22]. This pathway occurs widely in plant species, conferring adaptive advantages to diverse ecosystems. Its activation leads to the enhanced production of various phenylpropanoid compounds such as flavonoids [23,24], lignins [25], anthocyanins [26], salicylic acid [27], and stilbenes [28]. These metabolites reduce the adverse effects caused by stress-induced oxidative damage. One of the most studied stilbenes is *trans*-resveratrol. This compound is especially involved in plant–pathogen interactions [28] and plays an important role in plant responses to cadmium [29]. Besides the phenylpropanoid-based mechanism of plant responses to various biotic and abiotic stresses, another mechanism is the regulation of the ratio of S-containing compounds such as methionine, glutathione, phytochelatins, and glucosinolates by the activity of ATP-sulfurylase [30]. Through these S-compounds, that enzyme is involved in the plant tolerance of several biotic and abiotic stresses. For example, glutathione can control the gene expression of antioxidant enzymes such as superoxide dismutase or glutathione reductase as well as enzymes of the phenylpropanoid pathway (e.g., chalcone synthase and phenylalanine ammonia-lyase) under cadmium stress [31]. ATP-sulfurylase catalyzes the activation of SO_4_^2−^, yielding high-energy adenosine-5′-phosphosulfate (APS) [30]. It is known that in plants, APS can be converted into NH_2_-pNs by ammonolysis catalyzed by adenylylsulfate-ammonia adenylyltransferase [9] and Fhit proteins [5]. Moreover, Fhit can degrade NH_2_-pA, releasing AMP and NH_3_ [7,10,15].

The main goal of our research was to learn how NH_2_-pA as well as other NH_2_-pNs including NH_2_-pG (guanosine 5′-phosphoramidate), NH_2_-pC (cytidine 5′-phosphoramidate), and NH_2_-pU (uridine 5′-phosphoramidate) regulate the metabolism of phenylpropanoids and biosynthesis of phenylamides in grape cells. Moreover, we wanted to determine how these uncommon nucleotides impact the expression of the gene coding for the VvABCG44 transporter, which was proven to be involved in the transport (export) of *trans*-resveratrol in *Vitis vinifera*. This paper describes the results of experiments conducted on a *Vitis vinifera* cv. Monastrell suspension cell culture and presents a hypothesis concerning links between NH_2_-pNs and the production of *trans*-resveratrol.

## 2. Results

In this study, we used a suspension cell culture (SCC) of the grape cell cultivar Monastrell, which is a very convenient model. First, because of the equal distribution of molecules studied as effectors among the cells, and second, this particular variety of the grape effectively synthesizes the phenylpropanoid molecule *trans*-resveratrol. The analysis of gene expression and accumulation of different products of the phenylpropanoid pathway was carried out as described in our earlier studies [3,4,6]. For details, see Section 4. In our earlier studies on the effect of NH_2_-pA on the expression of the genes coding for phenylalanine ammonia-lyase (PAL) and 4-coumarate:coenzyme A ligase (4CL) in *Arabidopsis* seedlings, we found that of the concentrations tested in the 0.05–25 µM range, 5 µM NH_2_-pA appeared to be the most effective [4]. In addition, in the experiments on the grape suspension cells described here, this relatively low concentration of NH_2_-pA evoked marked effects. Therefore, each of the investigated NH_2_-pNs was applied to the cells at a fixed 5 µM concentration. Based on previous studies [3,4,6,32,33,34], we chose the following genes: *PAL1*, *C4H1*, *4CL1*, *STS1*, *CAD1,* and *CCR2*. In addition, we selected the time points of the experiment based on our previous works [3,6].

### 2.1. Do the NH_2_-pNs Affect Chalcone Synthase (CHS1) Gene Expression?

The expression of the *CHS1*, the branch point of flavonoid biosynthesis, was evaluated at the same time points as other gene expressions were analyzed in this study, but it was not detected. The same results were observed in the grape suspension cell culture in our previous studies [3,6] and by Lijavetzky and coworkers [32]. These data strongly suggest that flavonoids are not synthesized in the dark in cells of this plant species.

### 2.2. Effect of Exogenous NH_2_-pNs on the Expression of Genes of the General Phenylpropanoid Pathway

**Figure 3:**
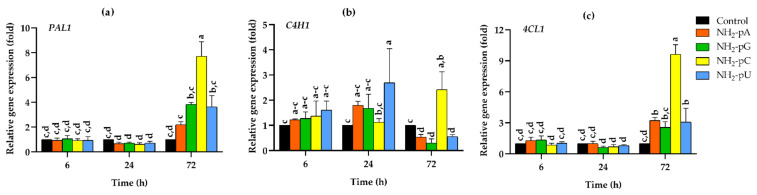
Expression of general phenylpropanoid pathway genes in cells of *Vitis vinifera* cv. Monastrell treated with 5 µM nucleoside 5′-phosphoramidates. (**a**) *PAL1*, phenylalanine ammonia-lyase; (**b**) *C4H1*, cinnamate-4-hydroxylase; (**c**) *4CL1,* 4-coumarate:CoA ligase. Total RNA was reverse-transcribed into cDNA and used as a template for real-time quantification PCR reaction as described in the Section 4. Specific primers were designed for *PAL1*, *C4H1*, *4CL1*, and *EFα1* (elongation factor 1-alpha, which was used as an endogenous control). The expression level of *PAL1*, *C4H1*, and *4CL1* in the control cells (no nucleotide added) was set to 1. Values represent the mean ± standard deviation of the three replicates. Values without a common letter were significantly different according to the analysis of variance (ANOVA) and Tukey’s honestly significant difference (HSD) multiple range test (*p* ≤ 0.05).

Expression of the three genes *PAL1*, *C4H1*, and *4CL1* was analyzed in the cells collected between 6 and 72 h of growth after elicitation. The results of these experiments are summarized in Figure 3a–c. A marked increase in the expression of the studied genes was observed in the grape cells collected after 72 h. Interestingly, NH_2_-pC evoked the most significant effect of the analyzed compounds, with an approximately 8-fold increase in *PAL1*. It was over 2-fold higher in comparison to the effect exerted by NH_2_-pG and NH_2_-pU and about 4-fold higher than that caused by NH_2_-pA. Additionally, the expression of *4CL1* was induced much more effectively by NH_2_-pC than by the other tested NH_2_-pN, and it reached about a 10-fold increase with respect to the controls. Effects evoked by 5 µM NH_2_-pG, NH_2_-pA, or NH_2_-pU were less spectacular, with only 4-, 3.2-, and 3-fold increases compared to the control, respectively. The expression of *PAL1* and *4CL1* after 6 and 24 h of elicitation by each of the tested nucleotides did not change. In the case of *C4H1* expression, we observed an inhibitory effect evoked by NH_2_-pU, NH_2_-pA, and NH_2_-pG at 72 h. It was about 2- to 3-fold lower than in the control. In cells treated with NH_2_-pC, the expression of *C4H1* increased up to 2.5-fold. However, it was 5- to 8-fold higher than in cells treated with other nucleotides (Figure 3a–c).

### 2.3. Effect of Exogenous NH_2_-pNs on Stilbene Synthase Gene (STS1) Expression and Stilbene Accumulation in Grape Cells

**Figure 4:**
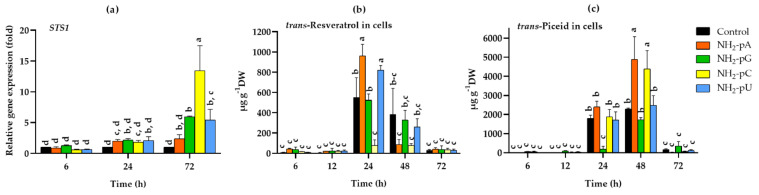
Expression of stilbene synthase gene (*STS1)* and accumulation of stilbenes in cells of *Vitis vinifera* cv. Monastrell treated with 5 µM nucleoside 5′-phosphoramidates. (**a**) *STS1*, stilbene synthase; (**b**) accumulation of *trans*-resveratrol; (**c**) accumulation of *trans*-piceid. Total RNA was reverse-transcribed into cDNA and used as a template for real-time quantification PCR reaction as described in the Section 4. Specific primers were designed for *STS1* and *EFα1* (elongation factor 1-alpha, which was used as an endogenous control). The expression level of *STS1* in the control cells (no nucleotide added) was set to 1. Values represent the mean ± standard deviation of the three replicates. Accumulation of *trans*-resveratrol and *trans*-piceid was determined using the HPLC method as described in the Section 4. Values without a common letter are significantly different according to ANOVA and Tukey’s HSD multiple range test (*p* ≤ 0.05).

All of the tested NH_2_-pNs increased the expression of *STS1* about 2-fold after 24 h, but only NH_2_-pG, NH_2_-pC, and NH_2_-pU also increased the expression of *STS1* in 72 h. At this time point, the most effective was NH_2_-pC, even causing a 13-fold higher expression than in the control (Figure 4a). Such a spectacular effect inspired us to investigate how this induction of *STS1* expression affects the accumulation of the related stilbene compounds (i.e., *trans*-resveratrol and its glycoside - *trans*-piceid). In the cells collected after 6 and 12 h, no significant effect of the tested nucleotides on *trans*-resveratrol content was found. However, after 24 h of elicitation with any of the investigated NH_2_-pNs, a dramatic increase in the level of this secondary metabolite was observed (Figure 4b). The most significant effect was evoked by NH_2_-pA and NH_2_-pU at 24 h. In their presence, the accumulation of *trans*-resveratrol in the grape cells reached 961 µg g^−1^ dry weight (DW) and 821 µg g^−1^ DW, respectively, and it was about 1.7- and 1.5-fold higher than in the control cells (Figure 4b). We did not observe statistically significant changes in *trans*-resveratrol accumulation in the presence of NH_2_-pG compared with the control cells. However, in grape cells treated with the pyrimidine nucleotide, NH_2_-pC, the accumulation of *trans*-resveratrol only reached 76 µg g^−1^ DW, and it was 7-fold lower than in the control cells. In the cells collected after 48 h, the *trans*-resveratrol content was much lower than that at 24 h of the experiment, and in those collected after 72 h, it was as low a level as in the cells collected after 6 and 12 h of elicitation (Figure 4b). The *trans*-piceid content, similar to *trans*-resveratrol, was low at 6 and 12 h, irrespective of the nucleotide treatment (Figure 4c). The content of *trans*-piceid was clearly elevated after 24 h of elicitation including in the control cells, but it was dramatically decreased in the cells treated with NH_2_-pG, being 13-fold lower than in the control cells and reached only 183 µg g^−1^ DW (Figure 4c). Interestingly, however, after 48 h, the accumulation of *trans*-piceid reached a maximum. At this time, in cells treated with NH_2_-pA or NH_2_-pC, the level of this stilbene was over 2-fold higher than in the control cells. Similar to the *trans*-resveratrol content, the level of *trans*-piceid decreased dramatically after 72 h of elicitation and reached a level comparable to that observed after 6 and 12 h of the experiment (Figure 4c).

### 2.4. Expression of the Gene Coding for the Resveratrol Transporter VvABCG44 (ATP-Binding Cassette Transporter) and Stilbene Content in the Spent Media

**Figure 5:**
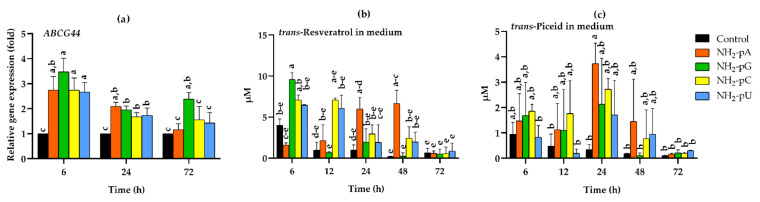
Expression of the *VvABCG44* resveratrol transporter gene in the cells of *Vitis vinifera* cv. Monastrell treated with 5 µM nucleoside 5′-phosphoramidates, and accumulation of stilbenes in the spent medium. (**a**) *VvABCG44*, resveratrol transporter gene; (**b**) accumulation of *trans*-resveratrol; (**c**) accumulation of *trans*-piceid. Total RNA was reverse-transcribed into cDNA and used as a template for real-time quantification PCR reaction as described in the Section 4. Specific primers were designed for *VvABCG44* and *EFα1* (elongation factor 1-alpha, which was used as an endogenous control). The expression level of *VvABCG44* in the control cells (no nucleotide added) was set to 1. Accumulation of *trans*-resveratrol and *trans*-piceid was determined using the HPLC method as described in the Section 4. Values are the mean ± standard deviation of the three replicates. Values without a common letter were significantly different according to ANOVA and Tukey’s HSD multiple range test (*p* ≤ 0.05).

It is known that treatment of cultured grape cells with an elicitor, cyclodextrin, causes the accumulation of *trans*-resveratrol and induction of gene expression of the full-size ABCG transporter, which is associated with the transport of this stilbene compound in plants [35]. Therefore, we also analyzed the effects of NH_2_-pNs on the expression of the *VvABCG44* gene. As shown in Figure 5a, each of the investigated nucleoside phosphoramidates evoked around a 3-fold increase of the gene expression, and it was already observed after 6 h of elicitation. Then, this gene expression declined at 24 and 72 h. An exception was observed in cells treated with NH_2_-pG at 72 h, since the increase in the gene expression was still 2-fold higher than in the controls. The time-course of *trans*-resveratrol and *trans*-piceid accumulation in the spent medium in response to NH_2_-pNs are shown in Figure 5b,c, respectively. Intensive export of these compounds from the cells to the media occurred, and it was observed just after 6 h of elicitation. The concentration of *trans*-resveratrol after 6 h of NH_2_-pG application really reached 9.5 µM (Figure 4b), but the concentration of *trans*-piceid after 6 h of NH_2_-pC reached less than 2 µM (Figure 5c). In the spent medium in which the cells were treated with NH_2_-pA, we observed a gradual increase in *trans*-resveratrol content up to 48 h. Then, the level of this stilbene drastically decreased (Figure 5b). At 72 h of the experiment, the concentration of *trans*-resveratrol was at the same level as in the control media. The highest accumulation of *trans*-piceid in the spent media caused by nucleotides was observed at 24 h, and for NH_2_-pA, NH_2_-pC, NH_2_-pG, and NH_2_-pU, it was 12-, 9-, 7-, and 5-fold higher than in the control media, respectively; however, only the 12-fold increase was statistically significant (Figure 5c).

### 2.5. Cell Viability

**Figure 6:**
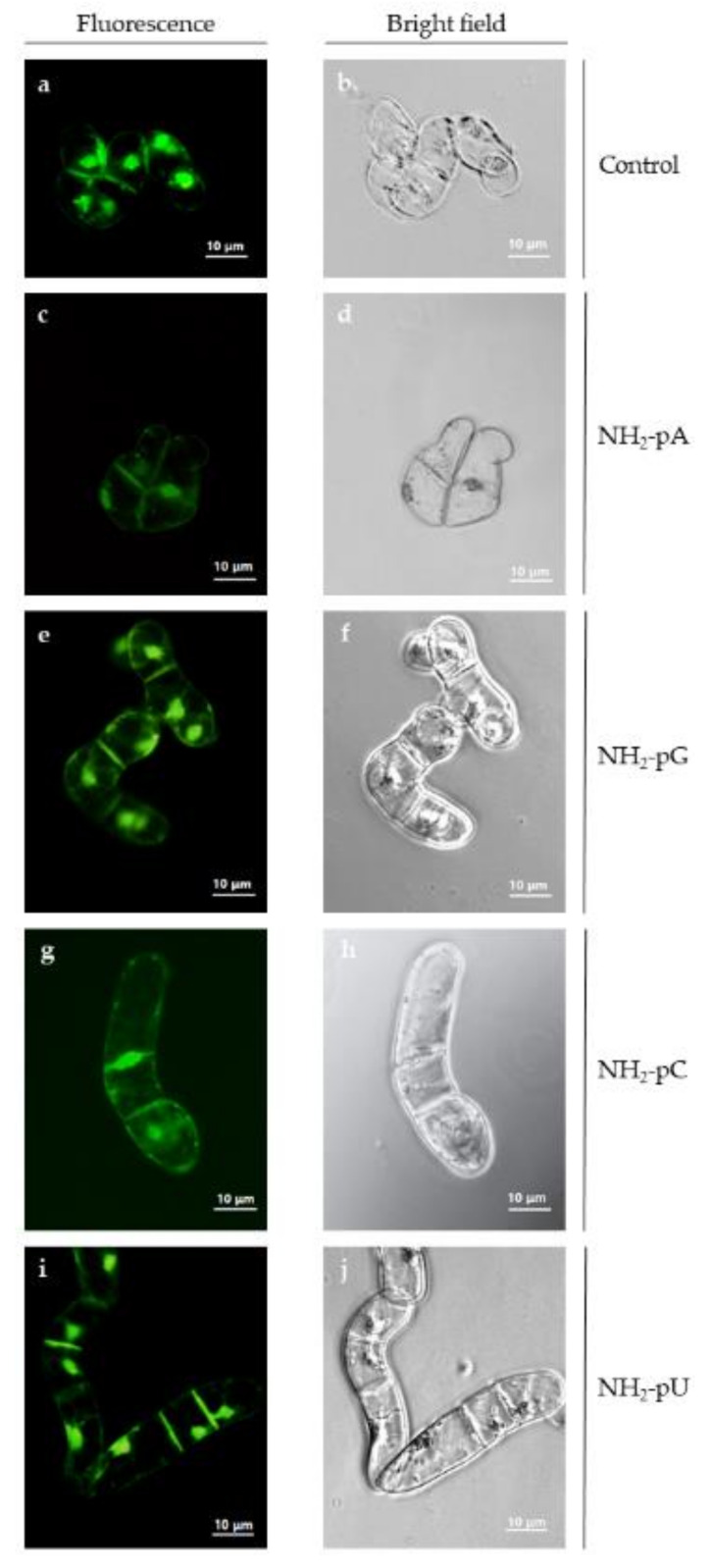
Viability of *Vitis vinifera* cell suspension culture treated with 5 µM NH_2_-pNs at 72 h. It was evaluated by incubating cells in the presence or absence of the indicated nucleotide for 1–2 min in fresh Gamborg medium using the Plant Cell Viability Assay Kit (Sigma-Aldrich, Burlington, MA, USA) as described in the Section 4. Fluorescence was observed with an AxioVert 200 Carl Zeiss microscope using a Zeiss filter (FS09 exc = 495 nm, emi = 517 nm). (**a**,**b**) control cells; (**c**,**d**) cells treated with NH_2_-pA; (**e**) and (**f**) NH_2_-pG; (**g**,**h**) NH_2_-pC; (**i**,**j**) NH_2_-pU. The left-hand column shows cells under the fluorescence microscope, and the right-hand column shows cells under the bright field microscope. No red fluorescence, which indicates cell damage or mortality (FS15 exc = 538 nm, emi = 617 nm) was found (data not shown).

Because we observed, both in the cells and in the spent media, a considerable decrease in the content of *trans*-resveratrol and *trans*-piceid at 72 h of the experiment, and due to the fact that at this time there was no effect of nucleotides on the content of these two stilbenes, we assessed the cell viability and cell growth (expressed as dry weight content) to exclude the possibility of cell death caused by the exogenous application of nucleotides. As shown in Figure 6, no losses in cell viability were observed by fluorescent microscopy at 72 h of treatment with the nucleotides.

**Figure 7:**
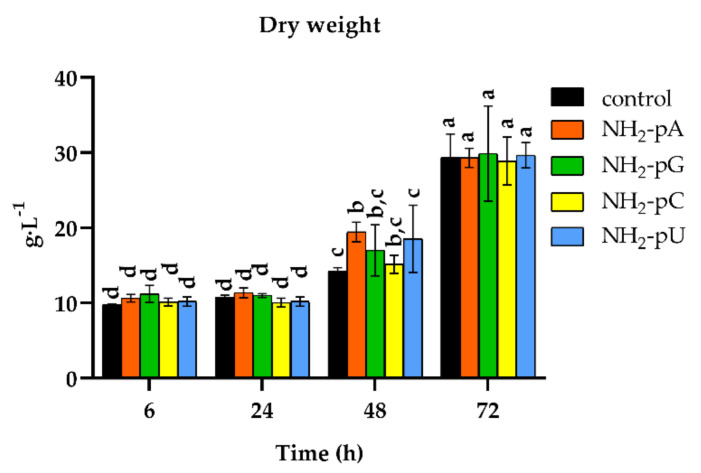
Time course of cell growth (expressed as cell dry weight per liter) of *Vitis vinifera* treated with 5 µM nucleoside 5′-phosphoramidates. Cell samples were air-dried for 48 h at 70 °C. Values represent the mean ± standard deviation of the three replicates. Values without a common letter were significantly different according to ANOVA and Tukey’s HSD multiple range test (*p* ≤ 0.05).

We also analyzed changes in the cell dry weight throughout the experiment. As shown in Figure 7, the nucleotide-treated cells displayed a similar biomass increase (from 10 to over 29 g DW L^−1^), and therefore cell growth as the control (i.e., untreated cells).

### 2.6. Expression of Genes Encoding for Enzymes of Monolignol Biosynthesis and Lignin Content

**Figure 8:**
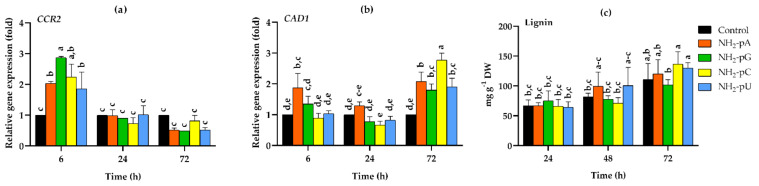
Expression of cinnamoyl-CoA reductase (*CCR2)*, cinnamyl alcohol dehydrogenase (*CAD1*), and lignin content in cells of *Vitis vinifera* cv. Monastrell treated with 5 µM nucleoside 5′-phosphoramidates. (**a**) *CCR2*, cinnamoyl-CoA reductase (**b**) *CAD1*, cinnamyl alcohol dehydrogenase (**c**) lignin content. Total RNA was reverse-transcribed into cDNA and used as a template for real-time quantification PCR reaction as described in the Section 4. Specific primers were designed for *CCR2, CAD1,* and *EFα1* (elongation factor 1-alpha, which was used as an endogenous control). The expression level of *CCR2* and *CAD1* in the control cells (no nucleotide added) was set to 1. The lignin content was determined as described in the Section 4. Values represent the mean ± standard deviation of the three replicates. Values without a common letter were significantly different according to ANOVA and Tukey’s HSD multiple range test (*p* ≤ 0.05).

We tested both the expression of genes involved in lignin biosynthesis and the accumulation of lignin, one of the main products of the phenylpropanoid pathway. *CCR2* gene encodes cinnamoyl-CoA reductase, which is the first step in monolignol biosynthesis. As can be seen in Figure 8a, the expression of *CCR2* was induced by nucleotides only at 6 h of treatment, and for NH_2_-pU, NH_2_-pA, NH_2_-pC, and NH_2_-pG, it was 1.8-, 2-, 2.2- and 2.9-fold higher than in the control, respectively. Then, a reduced expression of *CCR2* was observed, being at 72 h 2-fold lower in all nucleotide treatments than in the control (i.e., untreated cells) (Figure 8a).

*CAD1* gene expression, encoding cinnamyl alcohol dehydrogenase, was also assessed. Expression of this gene increased up to 2.7-fold in cells treated with 5 µM NH_2_-pC at 72 h. At this time point, all other tested nucleotides evoked only a 2-fold higher effect than in the control cells (Figure 8b).

On the other hand, the nucleotides investigated in this study during the experiments had no effect on the lignin content, and its level was similar to that found in control cells (Figure 8c).

### 2.7. Effect of Exogenous NH_2_-pNs on the Content of Phenylamides in Both Cells and the Spent Media

**Figure 9:**
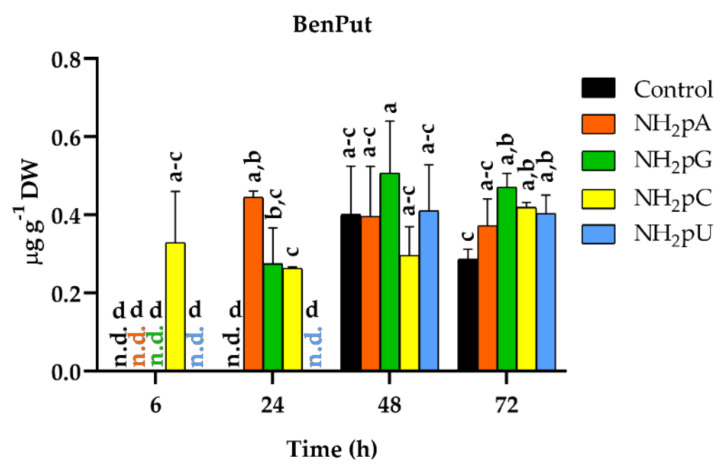
BenPut accumulation in cells of *Vitis vinifera* treated with 5 µM nucleoside 5′-phosphoramidates. Values represent the mean ± standard deviation of the three replicates. Values without a common letter were significantly different according to ANOVA and Tukey’s HSD multiple range test (*p* ≤ 0.05). n.d., not detected.

Among the twenty-five phenylamides tested (Supplementary Materials (Methods S2)), only *N*-benzoylputrescine (BenPut) was accumulated in grape cells. The content of this phenylamide depended on the NH_2_-pN nature and treatment time (Figure 9). Interestingly, at 6 h of treatment, NH_2_-pC evoked induction of accumulation of BenPut. Its content reached 0.32 µg g^−1^ DW, and its level hardly changed throughout the experiment. In the case of other NH_2_-pNs, BenPut was not detected at 6 h. At 24 h, an accumulation of BenPut was observed in cells treated with NH_2_-pA, NH_2_-pG, and NH_2_-pC. At the further time points of the experiment, both in the controls and cells treated with nucleotides, the level of BenPut was similar and remained stable. To our knowledge, this is the first time detecting the accumulation of phenylamides in *Vitis vinifera*.

On the other hand, we did not observe the accumulation of any of the twenty-five tested phenylamides in the spent media.

## 3. Discussion

This study demonstrated that the uncommon nucleotide NH_2_-pA, naturally occurring in organisms, applied to a cell suspension of *Vitis vinifera*, induced the expression of genes that control both the biosynthesis of stilbenes (Figure 4a and Figure 5a) and lignins (Figure 8a,b). This induction caused a transient accumulation of *trans*-resveratrol and *trans*-piceid, both in the cells and spent media (Figure 4b,c and Figure 5b,c), respectively. Another purine nucleotide, NH_2_-pG, also induced the gene expression of resveratrol-cell-membrane-transporter throughout the experiment (Figure 5a). In fact, three canonical congeners of NH_2_-pA: NH_2_-pG, NH_2_-pC, and NH_2_-pU, which have not been so far identified as natural metabolites in any organism, also affected the expression of the aforementioned genes and accumulation of stilbene compounds. Although all the tested nucleoside phosphoramidates acted as elicitors, some differences in effectiveness were observed among them. In fact, NH_2_-pA (purine nucleotide) proved to be the most effective in inducing *VvABCG44* gene expression as well as in *trans*-resveratrol and *trans*-piceid accumulation. Additionally, NH_2_-pC (pyrimidine nucleotide) turned out to be quite effective in inducing genes of the phenylpropanoid pathway in *Vitis vinifera*. However, during a short exposure time (6 h), NH_2_-pG evoked the most significant effect on the expression of *VvABCG44* (Figure 5a) and *trans*-resveratrol accumulation in the spent medium (Figure 5b) among all investigated NH_2_-pNs. The level of *trans*-resveratrol and *trans*-piceid decreased at 72 h of the experiment both in the medium and cells (Figure 4b,c and Figure 5b,c, respectively). As the cell suspension of *Vitis vinifera* remains alive at the end of the treatment with these nucleotides (Figure 6), it is plausible to think that *trans*-resveratrol and its glucoside, *trans*-piceid could be transformed by the action of cellular or extracellular peroxidases into other more complex stilbenes (such as viniferins) [36]. Results obtained from this study together with those previously carried out in *Arabidopsis* seedlings treated with NH_2_-pA [4] suggests that the investigated nucleotides can act as signal molecules in plants. Moreover, our earlier studies showed that any common nucleotides such as AMP, GMP, UMP, and CMP that could be a product of degradation of NH_2_-pNs did not evoke the accumulation of stilbenes in *Vitis vinifera* suspension cell culture [6].

We also investigated whether exogenously applied NH_2_-pNs affected in grape cells the biosynthesis of lignin - other compounds derived from the phenylpropanoid pathway, known to be accumulated in plant tissues in response to abiotic or biotic stresses [25]. It was found, however, that the nucleotides used substantially modified neither the lignin content nor the cell growth (assessed as cell dry weight). Still, it should be kept in mind that both lignin biosynthesis and dry weight accumulation are long-term processes, and 72 h of treatment might not be sufficient to observe this effect on the accumulation of lignin and cell dry weight. These results nevertheless suggest that NH_2_-pNs would be involved in the early signaling stages in response to environmental stimuli.

Considering the signaling role of the investigated nucleotides, the question is: what is the target of NH_2_-pA, or generally, all NH_2_-pNs? To answer this question, we postulate that in the control of gene expression by NH_2_-pNs, the HIT proteins, which catalyze the cleavage of the phosphoramide bond in these nucleotides, are involved. As mentioned earlier, hydrolysis of the P–N bond liberates more energy than splitting the phosphate anhydride (P-O) bond; −38 kJ/mol versus −34 kJ/mol, respectively [9]. Whether the postulated signal transduction mediated by NH_2_-pNs causes the adenylation, or generally the nucleotidylation, of the hypothetical target molecule, or causes only its conformational changes is another intriguing question awaiting elucidation. Speculating further, we suggest that, in plant cells, there is a link between the metabolism of sulfur and NH_2_-pAs: first, by the double role of Fhit protein, which can act as an adenylylsulfate:ammonia adenylyltransferase, and as nucleoside phosphoramidase [5]; and second, by the known activation of sulfate metabolic pathways under biotic and abiotic stresses in plants [30].

**Figure 10:**
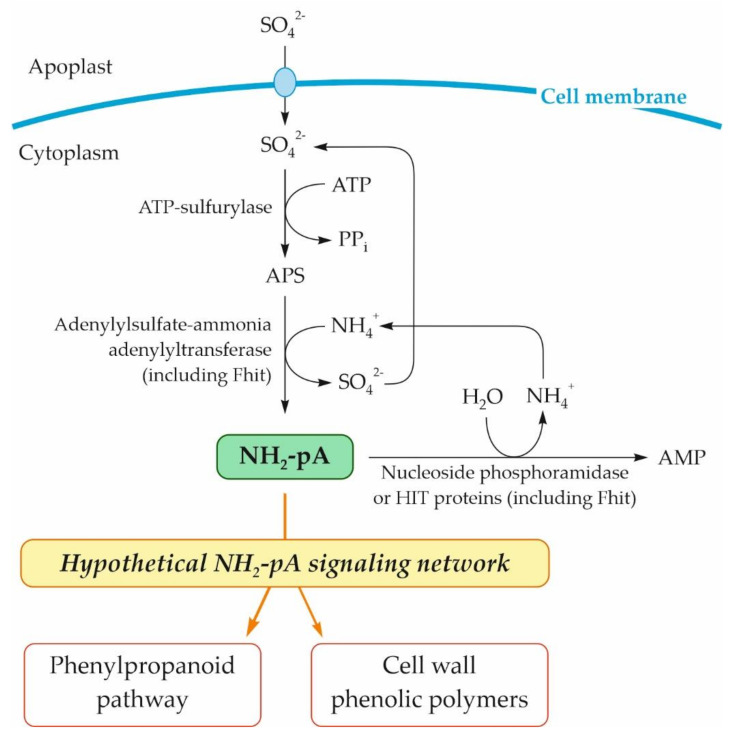
Schematic representation of metabolism of NH_2_-pA in plant cells and its effect on the phenylpropanoid pathway. APS, adenosine 5′-phosphosulfate.

Based on our previous studies on the effect of NH_2_-pA on phenylpropanoid metabolism in *Arabidopsis* seedlings [4], a fact considered in our literature review [37], and the results presented here, we postulate that NH_2_-pNs are involved in the plant response to environmental stresses via induction of the phenylpropanoid pathway. In Figure 10, we summarize the knowledge about the pathway of NH_2_-pA metabolism and its effect on the phenylpropanoid pathway. Although our results indicate that another NH_2_-pN (i.e., NH_2_-pC) exerts impressive effects on the same genes of the phenylpropanoid pathway (Figure 3 and Figure 4), we do not know if this compound occurs in nature and what enzymatic reaction might be responsible for its biosynthesis. We trust that our findings open new avenues that will be followed by different ’omic’ studies that will shed more light on physiological functions of these nucleotides.

## 4. Materials and Methods

### 4.1. Plant Materials

*Vitis vinifera* L. cv. Monastrell calli were established as described by Calderon et al. [38] and maintained at 25 °C in darkness in 250 mL flasks containing 100 mL of fresh culture medium (Gamborg B_5_, Duchefa, The Netherlands). Monastrell SCC was initiated by inoculating friable callus pieces in 250 mL Erlenmeyer flasks containing 100 mL of liquid Gamborg B_5_ medium (pH 6.0) at 25 °C in the dark and were routinely maintained by periodic subcultures every 14–16 days as described by Belchí-Navarro et al. [39] and Almagro et al. [40].

### 4.2. Elicitor Treatment

Elicitation experiments were carried out in triplicate using 10-day-old Monastrell SCC. At that stage of cell development, 3 g of fresh weight of cells was washed with cold distilled water, transferred into 50 mL flasks, suspended in 15 mL of fresh Gamborg B_5_ medium supplemented with 5 µM NH_2_-pN (NH_2_-pA, NH_2_-pG, NH_2_-pU, or NH_2_-pC), and incubated for 72 h at 25 °C in the dark on a rotary shaker (110 rpm). Control samples, without elicitors, were always run in parallel. The cells were harvested after 6, 24, 48, and 72 h, separated from the culture medium by filtration under a gentle vacuum, rapidly washed with cold distilled water, frozen in liquid nitrogen, and kept at −80 °C until use. The spent culture media were also frozen and stored at −20 °C until use.

### 4.3. NH_2_-pNs Chemical Synthesis

Details of the chemical synthesis of NH_2_-pA, NH_2_-pG, NH_2_-pU, and NH_2_-pC, and their characterization by HRMS, ^1^H NMR, ^13^C NMR, and ^31^P NMR are given in the Supplementary Materials (Methods S2).

### 4.4. Quantification of Trans-Resveratrol and Trans-Piceid

Extracellular content of *trans*-resveratrol and *trans*-piceid was determined as described by Pietrowska-Borek et al. [3,6]. For this, 20 µL of diluted and filtered (Anopore 0.2 µm) samples were analyzed by HPLC in the UV–VIS range using a LiChrospher 100 RP-18 column (250 × 4 mm, 5 µm; Merck, Darmstadt, Germany). Gradient elutions were performed with 0.05% TFA (solvent A) and 0.05% TFA in methanol:acetonitrile (60:40 *v*/*v*; solvent B): 0 min, 10% B; 5 min, 15% B; 40 min, 35% B; 45 min, 65% B; 50 min, 65% B; and 55 min, 10% B, setting the flow rate at 1 mL min^−1^. To determine the intracellular content of *trans*-resveratrol and *trans*-piceid, 200 mg of freeze-dried cells were extracted overnight with 4 mL of methanol at 4 °C with continuous shaking, and then 20 µL of each sample was analyzed on a LiChrospher 100 RP-18 column as described above. *trans*-Resveratrol and *trans*-piceid were identified (at 308 nm) and quantified by comparison with standards of commercial *trans*-resveratrol (Sigma-Aldrich, St. Louis, MO, USA) and *trans*-piceid (ChromaDex, Los Angeles, CA, USA) using respective calibration curves.

### 4.5. Lignin Determination

Lignin content was measured based on the method described by Syros et al. [41]. The harvested cells were air-dried at 70 °C, and 0.1 g dry mass was subjected to triple ethanol extraction at 80 °C. Each time, 3 mL of 80% (*v*/*v*) ethanol was added, and after the incubation, it was precisely discarded. The first extraction lasted for 1.5 h, the second, and the third for 1 h. Subsequently, 3 mL of chloroform was added, and the samples were heated to 62 °C. After 1 h, chloroform extract was removed, and samples were air-dried in an oven at 50 °C. Dried cells were digested at 70 °C in 2.6 mL of a solution of 25% (*v*/*v*) acetyl bromide in acetic acid containing 2.7% (*v*/*v*) perchloric acid. After 1 h of incubation, 100 µL of each sample was added to 580 µL of a solution of 2 N sodium hydroxide and acetic acid. The reaction was terminated by adding 20 µL of 7.5 M hydroxylamine hydrochloride. Then, the samples were filled up to 2 mL with acetic acid, and the absorbance at 280 nm was measured. Lignin content was expressed as mg g^−1^ DW, using a linear calibration curve with a commercial lignin alkali standard (Sigma, St. Louis, MO, USA).

### 4.6. Determination of Phenylamide Content in Cells and Spent Media

Phenylamide analysis was performed according to Morimoto et al. [19]. The grape cells were air-dried at 70 °C, then phenylamides were extracted with 10 mL of 80% methanol. For concentration, the samples were dried on a SpeedVac, and suspended in 300 µL of methanol. To extract phenylamides from the spent media, solid-phase extraction (SPE) (Superclean ENVI-18 SPE Tubes, Supelco, Bellefonte, PA, USA) was applied. The compounds from the SPE columns were eluted with 80% methanol and concentrated by drying on a SpeedVac, and suspending in 300 µL of methanol. Then, the samples were subjected to LC-MS/MS analysis. More details are given in the Supplementary Materials (Methods S2).

### 4.7. Cell Viability

Cell viability was evaluated using the Plant Cell Viability Assay Kit (Sigma-Aldrich) according to the manufacturer’s instructions. The cells were incubated for 1–2 min in fresh Gamborg medium, then 10 µL of the assay kit diluted in 1 M PBS pH 7.4 was added to 90 µL of cell suspension and mixed by gently tapping the tube. Fluorescence was monitored with an AxioVert 200 Carl Zeiss microscope using a Zeiss filter (FS09 exc = 495 nm, emi = 517 nm and FS15exc = 538 nm, emi = 617 nm).

### 4.8. Genes Expression Analyses

Total RNA was extracted from 200 mg of Monastrell frozen cells using the RNeasy Plant Minikit (Qiagen, Hilden, Germany) according to the supplier’s recommendations as previously described [3,6]. The concentration of each RNA sample was measured using a NanoDrop 2000 spectrophotometer (Thermo Scientific, Waltham, MA, USA). Only the RNA samples with a 260/280 ratio between 1.9 and 2.1 were used for the analysis. The integrity of RNA samples was also assessed by agarose gel electrophoresis and purity was confirmed by PCR using *EFα1*-specific primers. Then, 3 µg of total RNA was used for cDNA synthesis with oligo(dT)_20_ (50 µM) primers and the Superscript III Reverse Transcriptase Kit (Invitrogen). A quantitative real-time PCR reaction was carried out using a CFX96 Real-Time PCR Detection System (Bio-Rad) and iTaq Universal SYBR Green Supermix (Bio-Rad), and the specific primers for Monastrell genes (*PAL1*, *C4H1*, *4CL1*, *CHS1*, *STS1*, *VvABCG44*, *CCR2*, *CAD1*, and *EFα1*). The comparative *C_T_* method for relative quantification was used with *EFα1* as an endogenous control. The amount of target, normalized to an endogenous reference and relative to a calibrator, is given by 2^−∆∆CT^ [42]. Primer sequences and GenBank accession numbers are presented in the Supplementary Materials (Table S1).

### 4.9. Statistical Analysis

Data concerning mRNA level and concentrations of stilbenes, lignin, phenylamide and dry weight are the means of three independent replicates ± standard deviation. The statistical significance of the differences between averages was determined by ANOVA using Tukey’s HSD multiple range test at *p* ≤ 0.05.

## Data Availability

The data that support the findings of this study are available from the corresponding author upon reasonable request.

## Supplementary Materials

Supplementary Materials are available online at https://www.mdpi.com/article/10.3390/ijms222413567/s1.
