# Supplementary material for: Nucleoside 5′-Phosphoramidates Control the Phenylpropanoid Pathway in Vitis vinifera Suspension-Cultured Cells
Source: Int J Mol Sci. 2021 Dec 17;22(24):13567. doi: 10.3390/ijms222413567 (PMC8704414; doi:10.3390/ijms222413567)
Supplement: Supplementary file 1 [file ijms-22-13567-s001.zip › Supplementary files/Methods S1 NH2-pNs chemical synthesis.pdf]

Supplementary Materials

Nucleoside 5′-phosphoramidates control phenylpropanoid pathway in *Vitis vinifera* suspension-cultured cells

Methods S1: NH_2-pNs chemical synthesis

S1.1. Experimental

All reactions were performed in anhydrous solvents. Pyridine was dried over P_2O_5, distilled and kept over molecular sieves 4 Å until the amount of water was less than 20 ppm. The amount of water in solvents was determined with Karl Fisher coulometric titration. Solvents and volatile by-products were removed in a rotary evaporator under reduced pressure using water bath temperature not exceeding 40°C. NMR spectra were obtained on Bruker Avance II 400 MHz instrument in 5 mm tubes. For ^{31}P NMR experiments, 0.1 M solutions of the starting H-phosphonates were used. ^{31}PNMR chemical shifts are reported relative to 85% H_3PO_4 in water used as an external standard. Mass spectra were recorded on Thermo Fisher Scientific Q-Exactive Orbitrap mass spectrometer Brukermicro Q-TOF mass spectrometer with the ESI technique with negative ionization. For TLC analysis, the pre-coated plates (Merck silica gel F254) were used, and for column chromatography silica gel Si 60, 35-70 mesh (Merck) was used. Nucleoside 5′-H-phosphonates were obtained using a method developed by Romanowska et al.[1] Chemical reagents and solvents were commercial grade from Aldrich. Natural ribonucleosides (A, C, G, U) protected with the 2′,3′-di-*O*-acetyl group were obtained from ChemGenes. All synthesized compounds were of purity higher than 98% as judged from ^{1}H NMR spectroscopy.

S1.2. General procedure for the synthesis and purification of nucleoside 5′-phosphoramidates (NH_2-pNs)

Prior to the reaction, lithium amide (3 equiv. 153 mg) and the appropriate nucleoside 5′-H-phosphonate $TEAH^+$ salt (1 mmol, 1 equiv.) were separately dried overnight under vacuum. Nucleoside 5′-H-phosphonate was dissolved in pyridine (10 mL) and TMSCl (3 equiv., 381 μL) was added[2,3]. After 5 min, a lithium amide and then solution of I_2 in pyridine (1.5 equiv., 381 mg) were quickly injected into the reaction mixture. The mixture was stirred for 5 min at room temperature. After the reaction was complete (ca. 5 min, ^{31}P NMR), the mixture was quenched with addition of water and the excess of iodine was decomposed with ethanethiol. The solution was concentrated *in vacuo* and the residue was treated with aqueous ammonia (25%, 10 mL) for 1 hour. The products - NH_2-pNs were isolated by a silica gel column chromatography using a stepwise gradient of water (0–10% v/v) in acetonitrile containing Et_3N (5% v/v). Fractions containing pure products, appropriately NH_2-pA, NH_2-pG, NH_2-pC and NH_2-pU were collected and solvents evaporated to furnish colorless, crispy foams.

S1.3. General procedure for a direct conversion of nucleosides into nucleoside 5′-H-phosphonates

Ammonium (9H-fluoren-9-yl)methyl-H-phosphonate (0.5 mmol, 1.1 equiv.) was dissolved in a mixture of pyridine and triethylamine (in a ratio 4:1) and evaporated to convert it to the triethylammonium salt. To this, appropriate nucleoside (0.5 mmol, 1 equiv.) was added and both compounds were rendered anhydrous by repeated evaporation of the added pyridine. The residue was dissolved in 5 mL of CH_2Cl_2/pyridine (95:5, v/v) and PvCl (1.5 equiv.) was added. Phosphonylation was complete (^{31}P NMR, TLC) after ca 20 min. The reaction mixture was diluted with the same volume of methylene chloride, and washed with water (one third of the total volume) to afford H-phosphonate diesters. The organic layer was evaporated and the remaining oily residue was dissolved in CH_3CN/Et_3N [2 : 1, v/v; 5 mL per 0.5 mmol of diester] and kept for 20 min at room temperature to effect a quantitative elimination of the (9H-fluoren-9-yl)methyl group. The reaction mixture was evaporated and products were isolated by a silica gel filtration using a stepwise gradient of methanol (0 – 20% v/v) in methylene chloride containing Et_3N (3% v/v). Fractions containing pure product were collected and evaporated, to furnish colourless, crispy foams[1].

S1.4. Synthesis of adenosin-5′-yl phosphoramidate, TEAH⁺ salt (NH_2-pA)

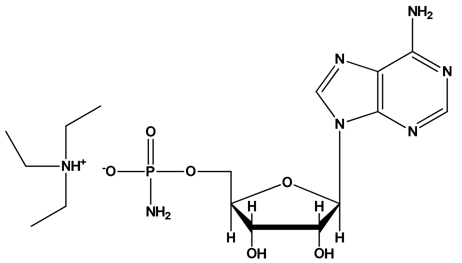

Adenosin-5′-yl phosphoramidate was obtained according to the general procedure starting from 2′,3′-di-*O*-acetyladenosine 5′-H-phosphonate $TEAH^+$ salt (mg, 1 mmol, 1 equiv.), and after purification by silica gel column chromatography compound was isolated as an triethylamonium salt (357 mg, 0.8 mmol, 80%).

Adenosin-5′-yl phosphoramidate, TEAH⁺ salt. Yield 80%. **HRMS *m/z*:** $[M-Et_3NH^+]^-$ calcd for $[C_{10}H_{14}N_6O_6P]^-$, 345.0718; found 345.0714. **^{1}H NMR** (D_2O, 400 MHz, δ): 8.43 (1H, s), 8.11 (1H, s), 6.06 (1H, d, $^3J_{H,H}$ = 5.6 Hz), 4.73 (1H, t, $^3J_{H,H}$ = 5.3 Hz), 4.50 (1H, m), 4.38 (1H, m), 4.10 (2H, m), 3.18 (6H, q, $^3J_{H,H}$ = 7.3 Hz), 1.26 (9H, t, $^3J_{H,H}$ = 7.3Hz). **^{13}C NMR** (D_2O, 100.6 MHz, δ): 154.84, 152.10, 148.62, 139.80, 118.23, 87.04, 83.94 (d, $J_{P,C}$ = 8.9 Hz), 74.30, 70.35, 63.85 (d, $J_{P,C}$ = 4.84 Hz), 46.59, 8.17. ^{31}P NMR{^{1}H} (CH_3CN, 121.4 MHz, δ): 9.3. **^{31}P NMR** (CH_3CN, 121.4 MHz, δ): 9.3 (m).

S1.5. Synthesis of guanosin-5′-yl phosphoramidate, TEAH⁺ salt (NH_2-pG)

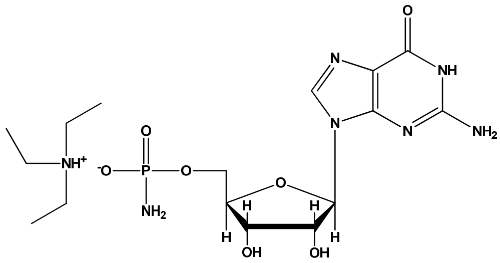

Guanosin-5′-yl phosphoramidate was obtained according to the general procedure starting from 2′,3′-di-*O*-acetylguanosine 5′-H-phosphonate TEAH$^+$ salt (mg, 1 mmol, 1 equiv.), and after purification by silica gel column chromatography compound was isolated as an triethylamonium salt (231 mg, 0.6 mmol, 50%).

Guanosin-5′-yl phosphoramidate, TEAH$^+$ salt. Yield 50%. **HRMS *m*/*z*:** [M-Et$_3$NH$^+$]$^-$ calcd for [$C_{10}H_{14}N_6O_7P$]$^-$, 361.0667; found 361.0660. **^{1}H NMR** (D_2O, 400 MHz, δ): 8.07 (1H, s), 5.88 (1H, d, $^3J_{H,H}$ = 5.6 Hz), 4.46 (1H, m), 4.31 (1H, m), 4.07 (1H, m), 3.17 (6H, q, $^3J_{H,H}$ = 7.3 Hz), 1.25 (9H, t, $^3J_{H,H}$ = 7.3 Hz). **^{13}C NMR** (D_2O, 100.6 MHz, δ): 157.12, 149.37, 147.90, 139.86, 119.86, 87.60, 84.16 (d, $J_{P,C}$ = 9.02 Hz), 73.50, 70.41, 63.95 (d, $J_{P,C}$ = 4.78 Hz), 46.63, 8.20. ^{31}P NMR{^{1}H} (CH_3CN, 121,4 MHz, δ): 9.4. ^{31}P NMR (CH_3CN, 121,4 MHz, δ): 9.4 (t, $^3J_{P,H}$ = 4.8 Hz).

S1.6. Synthesis of cytidine-5′-yl phosphoramidate, TEAH$^+$ salt (NH$_2$-pC)

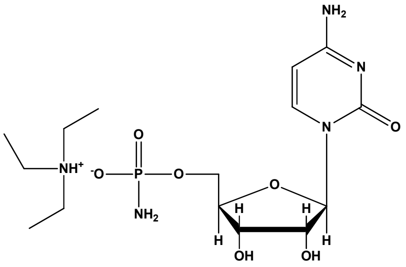

Cytidine-5′-yl phosphoramidate was obtained according to the general procedure starting from 2′,3′-di-*O*-acetylcytidine 5′-H-phosphonate TEAH$^+$ salt (mg, 1 mmol, 1 equiv.), and after purification by silica gel column chromatography compound was isolated as an triethylamonium salt (330 mg, 0.78 mmol, 78%).

Cytidine-5′-yl phosphoramidate, TEAH$^+$ salt. Yield 78%. **HRMS *m*/*z*:** [M-Et$_3$NH$^+$]$^-$ calcd for [$C_9H_{14}N_4O_7P$]$^-$, 321.0606; found 321.0614. ^{1}H NMR (D_2O, 400 MHz, δ): 8.00 (1H, d, $^4J_{H,H}$ = 7.6 Hz), 6.12 (1H, d, $^3J_{H,H}$ = 7.6 Hz), 5.98 (1H, d, $^3J_{H,H}$ = 2.7 Hz), 4.32 (2H, m), 4.18, 4.27 (1H, m), 4.17 (1H, m), 4.05 (1H, m), 3.20 (6H, q, $^3J_{H,H}$ = 7.3 Hz), 1.29 (9H, t, $^3J_{H,H}$ = 7.2 Hz). **^{13}C NMR** (D_2O, 100,6 MHz, δ): 165.48, 156.82, 141.66, 96.30, 89.38, 82.85 (d, $J_{P,C}$ = 8.9 Hz), 74.18, 69.35, 63.31 (d, $J_{P,C}$ = 4.7 Hz), 58.72, 46.61, 8.21. **^{31}P NMR**{^{1}H} (CH_3CN, 121,4 MHz, δ): 9.2. ^{31}P NMR (CH_3CN, 121,4 MHz, δ): 9.2.

S1.7. Synthesis of uridin-5′-yl phosphoramidate, TEAH$^+$ salt (NH$_2$-pC)

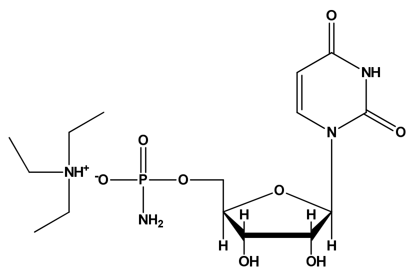

Uridin-5′-yl phosphoramidate was obtained according to the general procedure starting from 2′,3′-di-*O*-acetyluridine 5′-H-phosphonate $TEAH^+$ salt (mg, 1 mmol, 1 equiv.), and after purification by silica gel column chromatography compound was isolated as an triethylamonium salt (296 mg, 0.7 mmol, 70%).

Uridin-5′-yl phosphoramidate, $TEAH^+$ salt. Yield 70%. **HRMS *m/z*:** $[M\text{-}Et_3NH^+]^-$ calcd for $[C_9H_{13}N_3O_8P]^-$, 322.0446; found 322.0448. **^{1}H NMR** (D_2O, 400 MHz, δ): 8.00 (1H, d, $^4J_{H,H}$ = 8.2 Hz), 5.98 (1H, d, $^3J_{H,H}$ = 4.8 Hz), 5.95 (1H, d, $^3J_{H,H}$ = 8.1 Hz), 4.37 (1H, m), 4.34 (1H, m), 4.28 (1H, m), 4.12 (1H, m), 4.08 (1H, m), 3.21 (6H, q,$^3J_{H,H}$ = 7.3 Hz), 1.30 (9H, t, $^3J_{H,H}$ = 7.34 Hz). ^{13}C NMR (D_2O, 100.6 MHz, δ): 166.08, 151.67, 141.68, 102.46, 88.56, 83.40 (d, $J_{P,C}$ = 8.9 Hz), 73.82, 69.75, 63.54 (d, $J_{P,C}$ = 4.7), 46.62, 8.24. ^{31}P NMR{^{1}H} (CH_3CN, 121.4 MHz, δ): 9.2. **^{31}P NMR** (CH_3CN, 121.4 MHz, δ): 9.2.

References for supporting information:

1. Romanowska J., Szymanska-Michalak A., Pietkiewicz M., Sobkowski M., Boryski J., Stawinski J., Kraszewski A., A new, efficient entry to non-lipophilic H-phosphonate monoesters –preparation of anti-HIV nucleotide analogues, *Lett. Org. Chem.* 2009, 6, 496-499.
2. Bollmark M., Stawinski. J., A facile access to nucleoside phosphorofluoridate, nucleoside phosphorofluoridothioate, and nucleoside phosphorofluoridodithioate monoesters, *Tetrahedron Lett.* 1996, 37, 5739-5742.
3. Sun Q., Edathil J.P., Wu R., Smidansky E.D., Cameron C.E., Peterson B.R., One-pot synthesis of nucleoside 5'-triphosphates from nucleoside 5'-H-phosphonates, *Org. Lett.* 2008, 10, 1703-1706.
