# Supplementary material for: Nucleoside 5′-Phosphoramidates Control the Phenylpropanoid Pathway in Vitis vinifera Suspension-Cultured Cells
Source: Int J Mol Sci. 2021 Dec 17;22(24):13567. doi: 10.3390/ijms222413567 (PMC8704414; doi:10.3390/ijms222413567)
Supplement: Supplementary file 1 [file ijms-22-13567-s001.zip › Supplementary files/Methods S2 Identification and determination of content of phenylamides.pdf]

Supplementary Materials

Nucleoside 5′-phosphoramidates control phenylpropanoid pathway in *Vitis vinifera* suspension-cultured cells

Methods S2: Identification and determination of phenylamides content

The extracts of freeze dried cells and spent media were subjected to liquid chromatography coupled with tandem mass spectrometry (LC-MS/MS) in the multiple reaction monitoring (MRM) mode for the determination of phenylamide concentrations. Analysis was performed using a Quattro Micro API mass spectrometer (Waters, Milford, MA, USA) connected to an Acquity UPLC system (Waters). The LC conditions were as follows: column: ACQUITY UPLC BEH C18 (2.1 mm ID, 50 mm length, 1.7 µm particle size) (Waters); column temperature: 40°C; solvents: 0.1% formic acid in water (A) and 0.1% formic acid in acetonitrile (B); gradient: 5%-70% B/(A + B) within 10 min; flow rate: 0.2 mL/min. The MRM conditions were optimized using authentic compounds previously synthesized [1]. The ion transition, cone voltage and collision energy are summarized in Table 1. A mixture of authentic compounds in methanol at 10 µg/mL each were used for generation of standard curves.

1. Morimoto, N.; Ueno, K.; Teraishi, M.; Okumoto, Y.; Mori, N.; Ishihara, A. Induced phenylamide accumulation in response to pathogen infection and hormone treatment in rice (*Oryza sativa*). *Biosci. Biotechnol. Biochem.* **2018**, *82*, 407–416, doi:10.1080/09168451.2018.1429889.

Table. MRM conditions for the detection of phenylamides in cultured cells and spent media by liquid chromatography coupled with tandem mass spectrometry.

Compound*	Precursor ion (*m*/*z*)	Product ion (*m*/*z*)	Cone voltage (V)	Collision energy (eV)
CinAgm	261.19	130.92	25.00	32.00
CouAgm	277.11	146.89	25.00	26.00
CafAgm	293.09	162.83	30.00	30.00
FerAgm	307.05	176.89	34.00	24.00
BenAgm	265.21	175.93	25.00	20.00
CinTyr	268.12	131.00	20.00	14.00
CouTyr	284.10	146.87	25.00	22.00

CafTyr	300.09	162.82	20.00	22.00
FerTyr	313.99	176.91	28.00	18.00
BenTyr	242.20	120.98	20.00	26.00
CinTry	291.16	130.92	20.00	18.00
CouTry	307.08	146.93	20.00	22.00
CafTry	323.13	162.96	20.00	26.00
FerTry	337.04	176.90	30.00	14.00
BenTry	265.18	143.96	20.00	18.00
CinSer	307.14	130.91	20.00	22.00
CouSer	323.04	146.88	22.00	16.00
CafSer	338.97	176.95	22.00	12.00
FerSer	353.03	176.89	24.00	14.00
BenSer	281.16	160.04	20.00	16.00
FerPut	265.24	176.96	20.00	18.00
CafPut	251.19	162.97	20.00	22.00
CouPut	235.21	146.95	20.00	22.00
CinPut	219.23	102.93	20.00	34.00
BenPut	193.20	104.90	20.00	26.00

Abbreviations: CinAgm: *N*-cinnamoylagmatine; CouAgm: *N*-*p*-coumaroylagmatine; CafAgm: *N*-caffeoylagmatine; FerAgm: *N*-feruloylagmatine; BenAgm: *N*-benzoylagmatine; CinTyr: *N*-cinnamoyltyramine; CouTyr: *N*-*p*-coumaroyltyramine; CafTyr: *N*-caffeoyltyramine; FerTyr: *N*-feruloyltyramine; BenTyr: *N*-beyzoyltyramine; CinTry: *N*-cinnamoyltryptamine; CouTry: *N*-*p*-coumaroyltryptamine; CafTry: *N*-caffeoyltryptamine; FerTry: *N*-feruloyltryptamine; BenTry: *N*-benzoyltryptamine; CinSer: *N*-cinnamoylserotonin; CouSer: *N*-*p*-coumaroylserotonin; CafSer: *N*-caffeoylserotonin; FerSer: *N*-feruloylserotonin; BenSer: *N*-benzoylserotonin; FerPut: *N*-feruloylputresscine; CafPut: *N*-caffeoylputresscine; CouPut: *N*-*p*-coumaroylputresscine; CinPut: *N*-cinnamoylputresscine; BenPut: *N*-benzoylputresscine
