# Supplementary material for: Nucleoside 5′-Phosphoramidates Control the Phenylpropanoid Pathway in Vitis vinifera Suspension-Cultured Cells
Source: Int J Mol Sci. 2021 Dec 17;22(24):13567. doi: 10.3390/ijms222413567 (PMC8704414; doi:10.3390/ijms222413567)
Supplement: Supplementary file 1 [file ijms-22-13567-s001.zip › Supplementary files/Table S1. Primer sequences used for quantitative real-time polymerase chain reaction.pdf]

Table S1. Primer sequences used for quantitative real-time polymerase chain reaction.

Gene Symbol	GeneBank ID	Forward Primer (5′-3′)	Reverse Primer (5′-3′)	Amplicon Length (bp)
PAL1	XM_002281763.2	CCGAACCGAATCAAGGACTG	GTTCCAGCCACTGAGACAATC	183
C4H1	XM_002266202.1	TCCAAGTCACCGAGCCTGAT	GCAGGAATGTCATAGCCACC	109
4CL1	XM_002272746.2	CTGATGCCGCTGTTGTTTCG	GCAGGATTTTACCCGATGGA	198
CHS1	EC996578.1	GTCCCAGGGTTGATTTCCAA	TCTCTTCCTTCAGACCCAGTT	157
STS1	XM_002264419.2	CGCCAGGAGATAATCACTGCT	GCACCAGGCATTTCTACACC	134
CCR2	XM_002273418.3	ACAGCATGACGACTCTCTTCG	AGTGACAAGGGGTGGATTGA	182
CAD1	100247381	GGAGGGATGAAGGAGACACA	CTTTCAGCGTGTTGCCAATG	166
VvABCG44	AB910387.1	TAGGAGTGGTTGCAGCTGTG	TTTTGCTCCGTGTGACTTCTT	114
EFα1	XP_002284964.1	GAACTGGGTGCTTGATAGGC	AACCAAAATATCCGGAGTAAAAGA	164
